# Computational Analysis of KRAS Mutations: Implications for Different Effects on the KRAS p.G12D and p.G13D Mutations

**DOI:** 10.1371/journal.pone.0055793

**Published:** 2013-02-20

**Authors:** Chih-Chieh Chen, Tze-Kiong Er, Yen-Yi Liu, Jenn-Kang Hwang, Maria Jesus Barrio, Maximiliano Rodrigo, Enrique Garcia-Toro, Marta Herreros-Villanueva

**Affiliations:** 1 Institute of Bioinformatics and Systems Biology, National Chiao Tung University, Hsinchu, Taiwan; 2 Division of Molecular Diagnostics, Department of Laboratory Medicine, Kaohsiung Medical University Hospital, Kaohsiung, Taiwan; 3 Graduate Institute of Medicine, College of Medicine, Kaohsiung Medical University, Kaohsiung, Taiwan; 4 Department of Medical Laboratory Science and Biotechnology, Kaohsiung Medical University, Kaohsiung, Taiwan; 5 Servicio de Oncología, Hospital General Yagüe, Burgos, Spain; 6 Servicio de Anatomía Patológica, Hospital General Yagüe, Burgos, Spain; 7 Unidad de Investigación, Hospital General Yagüe, Burgos, Spain; Oak Ridge National Laboratory, United States of America

## Abstract

**Background:**

The issue of whether patients diagnosed with metastatic colorectal cancer who harbor KRAS codon 13 mutations could benefit from the addition of anti-epidermal growth factor receptor therapy remains under debate. The aim of the current study was to perform computational analysis to investigate the structural implications of the underlying mutations caused by c.38G>A (p.G13D) on protein conformation.

**Methods:**

Molecular dynamics (MD) simulations were performed to understand the plausible structural and dynamical implications caused by c.35G>A (p.G12D) and c.38G>A (p.G13D). The potential of mean force (PMF) simulations were carried out to determine the free energy profiles of the binding processes of GTP interacting with wild-type (WT) KRAS and its mutants (MT).

**Results:**

Using MD simulations, we observed that the root mean square deviation (RMSD) increased as a function of time for the MT c.35G>A (p.G12D) and MT c.38G>A (p.G13D) when compared with the WT. We also observed that the GTP-binding pocket in the c.35G>A (p.G12D) mutant is more open than that of the WT and the c.38G>A (p.G13D) proteins. Intriguingly, the analysis of atomic fluctuations and free energy profiles revealed that the mutation of c.35G>A (p.G12D) may induce additional fluctuations in the sensitive sites (P-loop, switch I and II regions). Such fluctuations may promote instability in these protein regions and hamper GTP binding.

**Conclusions:**

Taken together with the results obtained from MD and PMF simulations, the present findings implicate fluctuations at the sensitive sites (P-loop, switch I and II regions). Our findings revealed that KRAS mutations in codon 13 have similar behavior as KRAS WT. To gain a better insight into why patients with metastatic colorectal cancer (mCRC) and the KRAS c.38G>A (p.G13D) mutation appear to benefit from anti-EGFR therapy, the role of the KRAS c.38G>A (p.G13D) mutation in mCRC needs to be further investigated.

## Introduction

Colorectal cancer (CRC) is the third most common type of cancer worldwide [1] and the second leading cause of cancer deaths in the United States [2]. Recently developed therapies have significantly improved patient survival even after metastasis development. Despite these improvements in chemotherapy for metastatic colorectal cancer (mCRC), the overall five-year survival rate remains poor at only 11% for patients with metastatic disease [1]. Anti-epidermal growth factor receptor (anti-EGFR) therapies, involving cetuximab (Erbitux®, ImClone Systems) and panitumumab (Vectibix®, Amgen) have been approved by the US Food and Drug Administration (FDA) for the treatment of mCRC in the refractory disease setting [3], [4]. These monoclonal antibodies, chimeric and humanized, bind to the EGFR, preventing activation of the EGFR downstream signaling pathways, which are important for cancer cell proliferation, invasion, metastasis, and neo-vascularization [5]. One important member of this pathway is *KRAS*, and the evidence of anti-EGFR therapies improving the clinical benefits of wild-type (WT) *KRAS* in mCRC patients is well known and established [6], [7]. The *KRAS* gene encodes the KRAS protein that contains 188 amino acid residues with a molecular mass of 21.6kD [8]. KRAS is a membrane-associated GTPase that is an early player in many signal transduction pathways. KRAS acts as a molecular on/off switch to recruit and activate the proteins necessary for the propagation of the growth factor and other receptor signals, such as c-Raf and PI 3-kinase. When activated, KRAS is involved in the dephosphorylation of GTP to GDP, after which KRAS is turned off. However, the issue of whether patients harboring KRAS mutations can benefit from the addition of cetuximab or panitumumab to standard chemotherapy is under debate. Currently, health authorities in the United States and Europe have indicated that patients with *KRAS-*mutated tumors should not receive cetuximab or panitumumab. Consequently, only *KRAS* WT patients are treated with anti-EGFR therapies.

*KRAS* mutations are reported in approximately 40%–60% of all colorectal cancer specimens [9], [10]. These mutations principally occur in codons 12 and 13 and less frequently in codons 61, 63, and 146. Population-based studies have suggested that these mutations might be associated with some tumor phenotypes [11], [12]. Interestingly, recent study [13] indicated that the frequency of *KRAS* mutation was highest in caecal cancers among all subsites. The same group also proposed that luminal contents, including gut microbial communities and their metabolites, might trigger initiating molecular events or, alternatively, influence the tumour microenvironment and promote neoplastic progression [14].

When all these mutations are taken together, approximately 80% of patients have mutations in codon 12 whereas 18% have mutations in codon 13 [15]. Mutations in codons 61 and 146 have also been found to be oncogenic in KRAS, although these mutations occur at much lower prevalence (<5% of total *KRAS* mutations) than codon 12 and 13 mutations [16]. Mutations in codons 12 and 13 leads to alterations in encoded amino acids adjacent to the GDP/GTP binding pocket, reducing or abolishing the GTPase activity of KRAS after guanine nucleotide activating protein (GAP) binding and locking the protein in an active, GTP-bound state [17]. Both codons 12 and 13 in KRAS WT encode the glycine residues. The incorporation of other amino acids, most commonly aspartate and valine at codon 12 and aspartate at codon 13 [18], brings about the projection of larger amino acid side chains into the GDP/GTP binding pocket of the protein, interfering with the steric hindrance in GTP hydrolysis [19]. As a consequence, the EGFR signaling pathway is out of control with constitutive activation of the KRAS protein because of these conformational and structural changes.

With regard to anti-EGFR therapy, *KRAS* mutations conferring resistance to traditional anti-EGFR drugs have been reported [20], [21]. Many studies demonstrate that a small number of patients with *KRAS-*mutated tumors (10%) have responded to anti-EGFR therapy [22], [23] and approximately 15% have long-term disease stabilization [24]. In these patients, codon 13 mutations were overrepresented when compared with the overall *KRAS*-mutated tumor population. Additionally, *KRAS* codon 13 mutations might exhibit weaker *in* *vitro* transforming activity than codon 12 mutations [25], and some authors have indicated that *KRAS* codon 13 mutations may be associated with better outcomes after cetuximab treatment than with other *KRAS* mutations [26]–[29]. Nevertheless, the molecular mechanism leading to this outcome remains unknown. Recently, Tejpar et al. demonstrated that the addition of cetuximab to first-line chemotherapy appears to benefit patients with *KRAS* c.38G>A (p.G13D) tumors, and the relative treatment effects were similar to those in patients with KRAS wild-type tumors but with lower absolute values [30]. A small number of available experimental data demonstrate that tumor clones carrying *KRAS* codon 13 mutations are less aggressive than those carrying codon 12 mutations. This is because *KRAS* codon 13 exhibit higher levels of apoptosis [31]. Several studies have suggested that there is a reduced transforming capacity of the codon 13 mutation compared with the codon 12 mutation tested in both in vitro and in vivo systems [32], [33]. On the contrary, Tveit et al. [34] and Gajate et al. [35] did not observe any difference in the efficacy of cetuximab when comparing the codon 13 with codon 12 mutations. Moreover, *KRAS* codon13-mutated mCRCs were classified as poor prognostic markers and more aggressive in several studies [36]–[38].

Because of the lack of a consensus about whether *KRAS* mutated in codon 13 can confer different CRC phenotypes or responses to anti-EGFR therapies, there remains a need to clarify the molecular mechanisms underlying the changes occurring in the structure of the protein because of the different mutations. To address these questions, we employed a series of simulations to study the molecular mechanisms of c.35G>A (p.G12D), c.38G>A (p.G13D), and WT. We sought to test the hypothesis that a single residue substitution on codon 13 of KRAS could have effects on its dynamics, and a simple amino acid substitution might influence the structural dynamics of KRAS and hence its affinity to ligands and finally these changes would affect the patient’s response to the treatment.

## Materials and Methods

### Molecular Modeling

A (PS)^2^ server [39], [40] was used for building the homology-based models. The server uses effective consensus strategies, combining structural- and profile-based comparison methods, for both template selection and target-template alignment. For this study, the (PS)^2^ server selected the X-ray crystal structure of the KRAS-GTP complex (PDB ID: 3GFT) through a template consensus strategy [40] as the template structure. The models of WT KRAS and MT KRAS (c.35G>A (p.G12D) and c.38G>A (p.G13D)) were built using this template. Finally, the KRASGTP complexes were constructed by superimposing the predicted KRAS models on to the crystal structure of the KRAS-GTP complex.

### Molecular Docking

The modeling structures were used as the initial coordinates for docking purposes. The binding site for virtual docking was determined by considering the protein residues located ≤8 Å away from the GTP binding pocket. iGEMDOCK v2.1 [41], [42] was used to generate the docked conformation of ligands and to rank the conformations according to their docking scores. We used its molecular docking platform to dock the GTP to the active cavity of the KRAS models (WT and MT) with a population size of 300, a number of generations of 80, and the number of solutions set to 100. These 100 docking scores were then used for statistical analysis to evaluate the binding affinity between the KRAS models and GTP.

### Molecular Dynamics

MD simulations were performed using the GROMACS package with the GROMOS96 43A1 force field [43]. The topology files for the ligands were obtained from the PRODRG server [44]. The systems were solvated with simple point charge (SPC) water molecules, and the systems were simulated in a cubic box with periodic boundary conditions. The energy of the systems was first minimized using the steepest descent algorithm until it reached a tolerance of 10 kJ/mol/nm. After equilibrating with fixed protein at 300 K for a number of picoseconds, all of the systems were gradually relaxed and heated up to 300 K. Finally, the MD simulations were performed under constant pressure and temperature for 20.0 ns using an integration time step of 2 fs. Additionally, the electrostatic interactions were calculated using the PME algorithm [45] with an interpolation order of 4 and a grid spacing of 0.16. The non-bonded interactions were cutoff at 14 Å. The coordinates from the MD simulations were saved every 2 ps. The analyses were performed using the programs within the GROMACS package. The 3D molecular graphs were displayed using PyMOL [46].

### Analysis of MD Trajectories

The trajectories of WT and MT were analyzed for the following structural properties as a function of time: (a) the root mean square deviation (RMSD) of the sensitive sites (P-loop, switch I and II regions) with respect to their starting conformations; (b) the pocket distances between the mass center of residues 12–13 and the mass center of residues 32–34, which are located at the P-loop and switch I region, respectively; (c) the B-factors [47] of Cα atoms, which were calculated from the last 10.0 ns of the MD trajectories; (d) the covariance analysis of Cα atoms. The RMSD is the measure of the average distance between the atoms of the superimposed proteins. Therefore, it can be used to evaluate the degree of protein conformational change. The B-factors in the protein structures reflect the fluctuation of atoms about their average positions. A large B-factor indicates high flexibility of the individual atoms.

### Potential of Mean Force (PMF) Simulations

To explore the free energy profiles for the process of GTP binding with wild-type KRAS and its mutants (c.35G>A (p.G12D) and c.38G>A (p.G13D)), PMF simulations were performed using umbrella-sampling MD simulations [48]. The PMF is defined as the potential that gives an average force over all the configurations of a given system [49]. It generated a series of configurations along a reaction coordinate, after which umbrella-sampling was used to restrain these conformations within the sampling windows.

The total number of windows for each complex structure was approximately 30, depending on the initial structure of each system. Each window was separated by 1.0 Å, covering the reaction coordinates from ∼8 Å to 40 Å. The biasing force constant applied in the different windows of the umbrella sampling was 10.0 kcal/(mol.Å^2^). The selected conformations for each window were first equilibrated for 100 ps and then kept for 1 ns for production sampling. The frequency of the data collection was set to 1 ps, which was identical to that of the time step of umbrella-sampling MD.

After the umbrella-sampling MD simulations were finished for each system, the data collected from the separate simulation frames were combined along the reaction coordinates. These data were then used to calculate the PMF for the entire binding process using the weighted histogram analysis method (WHAM) [50], [51].

## Results

### Molecular Modeling and Structural Analysis of Human KRAS

**Figure 1:**
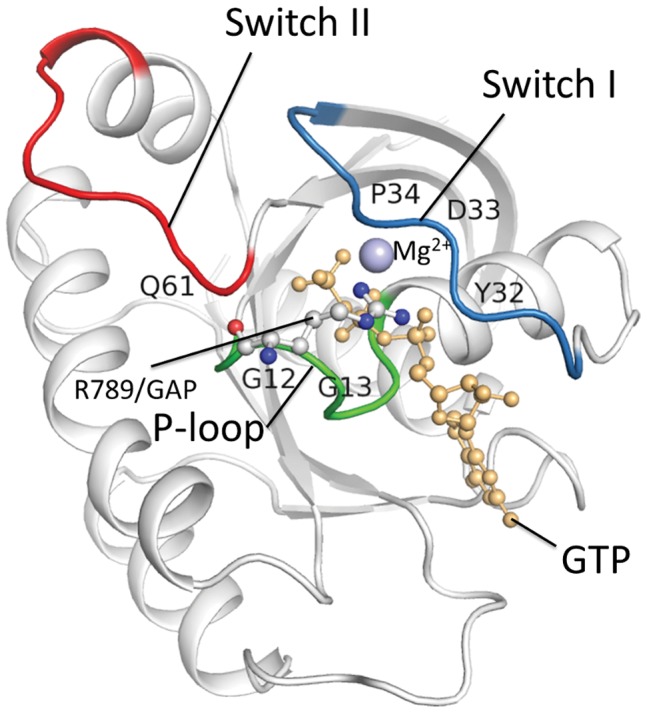
Molecular modeling of Human KRAS. The structure contains three sensitive sites: the P-loop (green), the switch I region (blue) and the switch II (red). The GTP and the Mg^2+^ ion are shown by ball-and-stick representations.

Human KRAS-GTP models were constructed using the published crystal structure (PDB Id: 3GFT) as the template (**Figure 1**). The sensitive sites were located at the regions that participate in the GTP hydrolysis. This includes the P-loop (phosphate-binding loop, amino acids 10–16), which binds the γ-phosphate of GTP, and switch I (amino acids 32–38) and II (amino acids 60–67), which regulate binding to the KRAS regulators and effectors. The amino acid R789/GAP is an important catalytic residue that interacts with the P-loop. The KRAS mutations of p.Gly12Asp and p.Gly13Asp were constructed using the same method as the WT structure and the replacements were both located in the P-loop region. The aim of this study was to perform a detailed examination of the structural flexibility of the P-loop and the switch I and II regions of human KRAS upon its binding with GTP.

### Protein Dynamics Simulation Analysis

**Figure 2:**
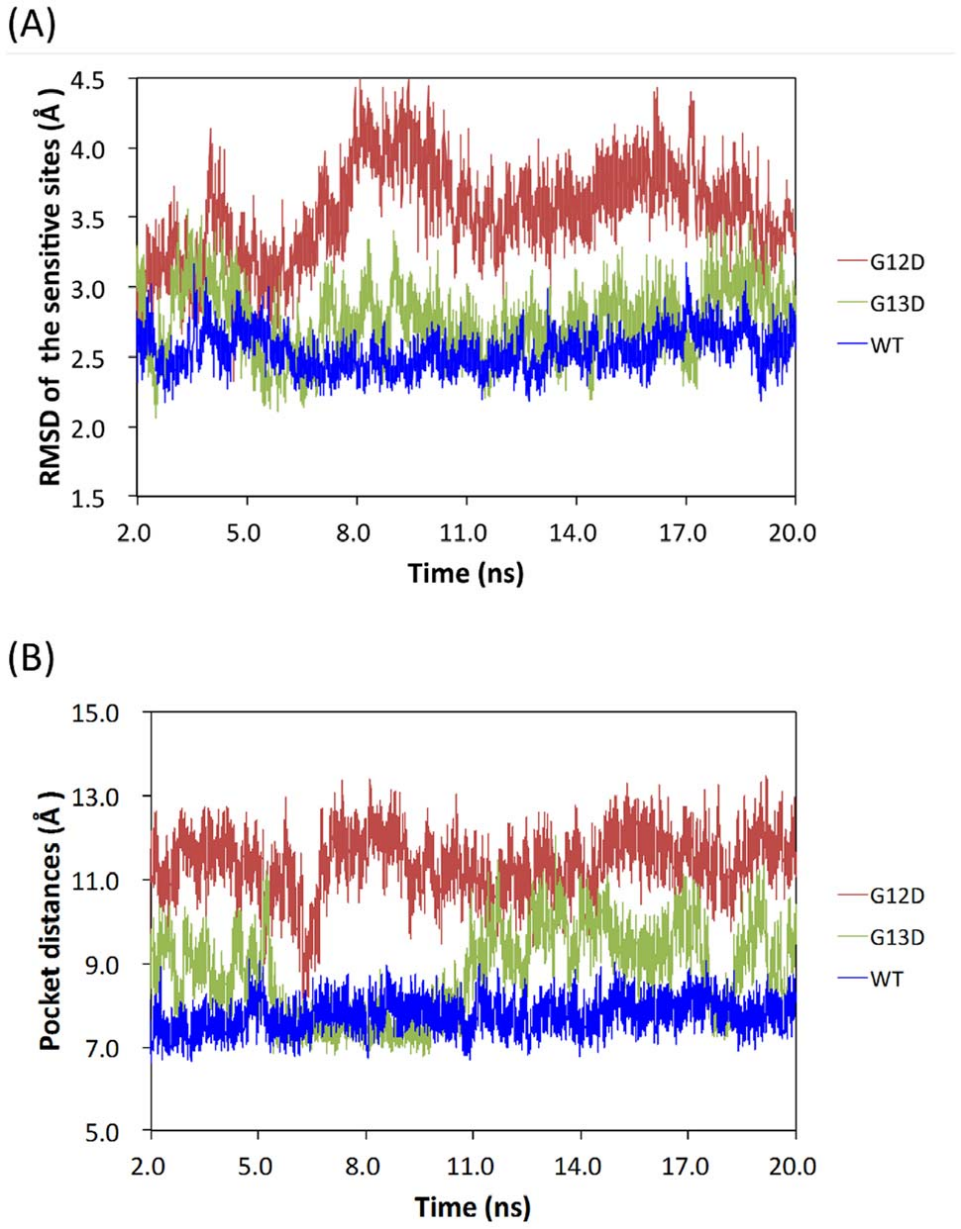
The molecular dynamics trajectories for: (A) Comparison of the RMSD plots of the sensitive sites (P-loop, switch I and II regions) of WT, G12D and G13D structures with respect to the initial conformation during the course of the simulation; (B) the pocket distances between the mass center of residues 12–13 and the mass center of residues 32–34 for WT, G12D, and G13D, respectively.

**Figure 3:**
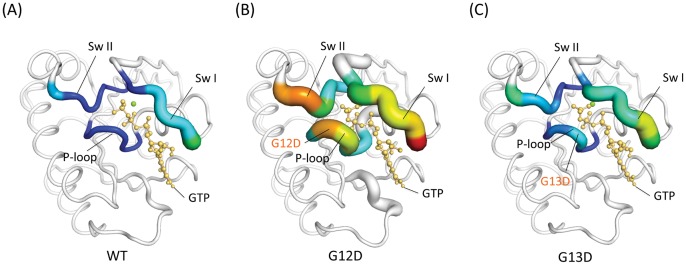
Analysis of atomic fluctuations. The structures of (A) WT, (B) G12D and, (C) G13D KRAS proteins are drawn in cartoon putty representations at the P-loop, switch I and II regions; blue represents the lowest and red the highest B-factor value. In addition, the size of the tube reflects the value of the B-factor, in that the larger the B-factor, the thicker the tube. The structures in the other regions are colored in white and displayed in cartoon tube representation, where the size of the tube is independent of the B-factors.

For the MD simulations, the trajectories of the WT and MT KRAS in the explicit solvent were calculated. The backbone RMSD values for WT and MT KRAS during the production phase relative to the starting structures were plotted (**Figure S1**) to obtain an estimate of the MD trajectory quality and convergence. The simulations of WT and MT KRAS indicate that, after a rapid increase during the first 2.0 ns, the trajectories stabilized, with average values of 1.54 Å, 1.82 Å, and 1.61 Å for WT and the c.35G>A (p.G12D) and c.38G>A (p.G13D) KRAS mutants, respectively. Statistical analysis of the RMSD data reveals that the trajectories are more stable after the first 10 ns. Therefore, only the second half of the trajectory was analyzed for the B-factor calculation. The RMSD of the sensitive regions with respect to the starting conformation was compared for the WT and MT structures during the course of the simulations. The RMSD was found to increase as a function of time for MT c.35G>A (p.G12D) when compared with WT and MT c.38G>A (p.G13D) (**Figure 2A**). By monitoring the pocket distances between the mass center of residues 12–13 and the mass center of residues 32–34, we found that the GTP-binding pocket in the c.35G>A (p.G12D) protein was more open than that of the WT and c.38G>A (p.G13D) proteins (**Figure 2B**). The results of calculating the B-factors for each residue at the sensitive sites (P-loop, switch I and II regions) revealed that the atomic fluctuations of c.35G>A (p.G12D) mutant were significant at the switch II and P-loop regions when compared with the WT and the MT c.38G>A (p.G13D) (**Figure 3**). In addition, the covariance matrix between residues represented by their Cα atoms was calculated for MD trajectories. It is evident from the three covariance matrices (**Figure S2**) that the c.35G>A (p.G12D) differ markedly from the WT and c.35G>A (p.G13D) and display increased levels of motional correlation (red) and anti-correlation (blue). This is consistent with the fluctuations calculated for the c.35G>A (p.G12D), which demonstrated that the amplitude of the fluctuations is larger than the WT and c.38G>A (p.G13D) structures. The data from the MD simulations on the c.35G>A (p.G12D) mutant GTP complex suggest that the c.35G>A (p.G12D) mutation leads to a considerable decrease in the affinity for the mutant enzyme binding with GTP. In order to make our studies more convincing, two additional molecular dynamics simulations with different initial velocities were performed. These results also agree with our results mentioned above (**Figures S3 and S4; Figures S5 and S6**).

### GTP-binding Analysis

**Figure 4:**
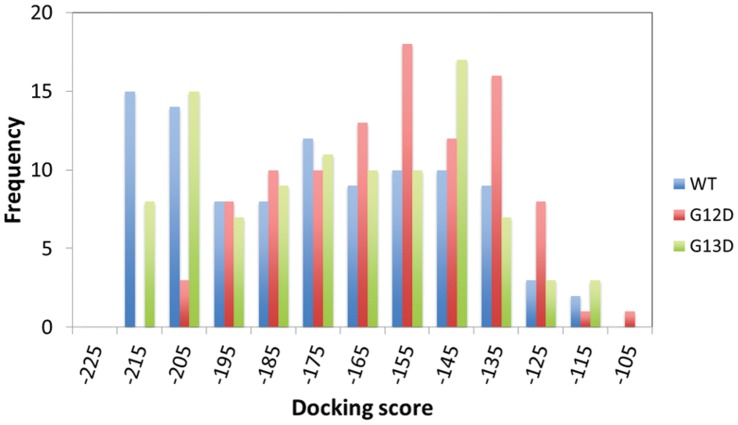
Distributions of docking scores. The docking scores for WT (blue), G12D (red) and G13D (green) KRAS proteins docked with GTP are shown in different colors.

Molecular docking is a widely used computational tool for the study of molecular recognition, which aims to predict the binding mode and affinity of a complex. **Figure 4** displays the distribution of the docking scores for WT, c.35G>A (p.G12D) and c.38G>A (p.G13D) docked with GTP. These experimental results demonstrate that WT (blue) and c.38G>A (p.G13D) (green) have lower (better) docking score distributions than c.35G>A (p.G12D) (red). The average of the docking scores are −180.27, −163.16 and −175.75 for WT, c.35G>A (p.G12D) and c.38G>A (p.G13D), respectively. Based on student’s t-test, WT (p-value is 5.9E−6) and c.38G>A (p.G13D) (p-value is 6.1E−4) have significantly better docking scores than c.35G>A (p.G12D).

**Figure 5:**
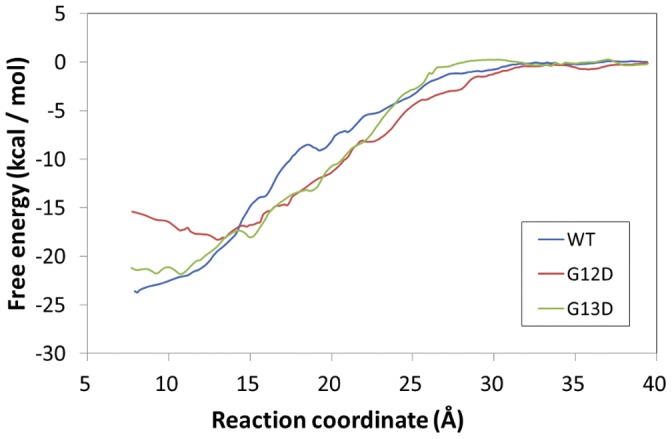
Free energy profiles determined for the binding of GTP with WT (blue), G12D (red) and G13D (green). The reaction coordinate is defined as the distance between the mass center of the GTP and the mass center of the WT, G12D, and G13D KRAS proteins, respectively.

To predict the binding free energy of GTP with wild-type KRAS and its mutants (c.35G>A (p.G12D) and c.38G>A (p.G13D)), the PMF simulations were performed starting from the MD-simulated KRAS-GTP complex structures. **Figure 5** depicts the PMF-calculated free energy profiles. The calculated binding free energy (ΔG_bind_) is −23.0 kcal/mol for wild-type KRAS, −14.6 kcal/mol for the c.35G>A (p.G12D) mutant, and −20.1 kcal/mol for the c.35G>A (p.G12D) mutant. The difference in the binding free energy between the p.Gly13Asp mutant and wild-type KRAS, that is, ΔΔG_bind_ = ΔG_bind_ (mutant) – ΔG_bind_ (wild-type), is 2.9 kcal/mol. The calculated ΔΔG_bind_ for the difference between the c.35G>A (p.G12D) mutant and the wild-type KRAS is 8.4 kcal/mol. According to the calculated relative binding free energies, GTP should have a much lower binding affinity with the c.35G>A (p.G12D) mutant than with wild-type KRAS or the c.38G>A (p.G13D) mutant, and the order of the binding affinity is wild-type KRAS>c.38G>A (p.G13D) mutant>c.35G>A (p.G12D) mutant.

## Discussion

The frequency of *KRAS*-mutated patients in a European cohort was 47% (**Figure S7A**). The total percentage of patients with c.38G>A (p.G13D) mutations was 18.6% (**Figure S7B**) and 8.73% when considering the entire CRC population (**Figure S7A**). However, this frequency may differ between populations; for instance, in the Taiwanese population, this frequency ranged from 33% to 40% [52], [53]. Such population differences could be because of detection limits of the methodologies used and the homogeneity of specimens. However, taking together different international published series, mutations in c.35G>A (p.G12D) and c.35G>T (p.G12V) were reported to be the most frequent and c.34G>A (p.G12S), c.35G>C (p.G12R) and c.34G>C (p.G12R) were reported to be the least frequent [54], [55]. Moreover, mutations in codon 13, in which the glycine changes to aspartate, account for over 80% of the mutations occurring in this codon and approximately 19% of *KRAS* mutations in patients [55], [56]. Similarly, the prevalence of *KRAS* mutations in codon 12 and 13 was 79% and 21%, respectively, in the Taiwanese population (unpublished data, total 420 CRC patients). Because the mutations in *KRAS* codon 13 represent a high proportion of the population suffering from CRC, the issue regarding the possible response of this mutated form of KRAS to anti-EGFR therapies should be considered relevant from biological, ethical and economical perspectives.

Different *KRAS* mutations in codon 12 could be associated with distinctive clinicopathological feature. Clinical studies shows that the *KRAS* codon 12 mutation, especially the c.35G>T (p.G12V) mutation, was associated with the highest colorectal cancer–specific mortality [25]. However, an experimental study showed that c.34G>C (p.G12R) and c.35G>T (p.G12V) mutations confer more potent transforming ability than other *KRAS* mutations, including c.34G>A (p.G12S), c.34G>T (p.G12C), c.35G>A (p.G12D), and c.35G>C (p.G12A) [17]. Furthermore, the GTPase activity of c.34G>C (p.G12R) and c.35G>T (p.G12V) mutants is lower than that of other *KRAS* mutations [57], [58]. Previously, MD calculations were performed on the wild-type p21^ras^, the p.G12V oncogenic mutant p21^ras^, and the p.G12P non-oncogenic mutant p21^ras^ [59]. The results indicated that the structure of the active site of the enzyme substrate complex in the oncogenic mutant p21^ras^ continuously changes, and these changes in the active site would make it difficult for the GTPGDP hydrolysis reaction to occur in the mutant. Recently, Messner et al. [60] indicated that p.G13D mutated CRC cells are more sensitive to anti-EGFR treatment than codon 12-or codon 61-mutated cells and the p.G13D-mutated CRC cells seem to define a less aggressive phenotype. Similarly, De Roock W et al. [26] suggested that p.G12V-mutated cells were insensitive to cetuximab, however, p.G13-mutated cells were nearly as response to cetuximab as wild-type cells.

The rate of GTP-to-GDP conversion can be dramatically accelerated by an accessory protein of the guanine nucleotide activating protein (GAP) class, for example, RasGAP [61]. KRAS undergoes conformational changes when it binds GTP. This binding involves two regions of the protein–(1) the switch I region and (2) the switch II region–which together form an effector loop that is responsible for controlling the specificity of the binding of GTPase to its effector molecules. This conformational change in the KRAS protein affects its interactions with multiple downstream transducers, that is, the GTPase-activating protein (GAPs) that amplify the GTPase activity of KRAS [62]. In the current study, our results revealed that the conformational changes of the c.35G>A (p.G12D) mutant were significant at these sensitive sites when compared with the WT and the MT c.38G>A (p.G13D) (**Figure 2**). Moreover, the mutation of c.35G>A (p.G12D) may also induce additional fluctuations at these sensitive sites (**Figure 3**). As mentioned earlier, the switch regions I and II play important roles in the binding of regulators and effectors; therefore, we postulate that such fluctuations may promote instability in both the regions, which consequently influences the binding ability of GTPase to its effector molecules and interferes with the interactions with GAPs. As a result, impairment of the GTPase activity leads to the active form of KRAS.

It should be noted that the incorporation of other amino acids in codons 12 and 13 in WT KRAS, most commonly aspartate and valine at codon 12 and aspartate at codon 13 [18], brings about the projection of larger amino acid side chains into the GDP/GTP binding pocket of the protein, thereby interfering with the steric hindrance in GTP hydrolysis [19]. Indeed, our results demonstrated by monitoring the pocket distances between the mass center of residues 12–13 and the mass center of residues 32–34 that the GTP-binding pocket in the c.35G>A (p.G12D) mutant is more open than that of the WT and c.38G>A (p.G13D) proteins (**Figure 2B**). According to the molecular docking and PMF simulations for the c.38G>A (p.G13D) mutant-GTP binding, the distribution of docking scores (**Figure 4**) and the simulated free energy profile (green curve in **Figure 5**) are also similar to that of the wild-type KRAS-GTP binding. The data obtained from the molecular docking, MD and PMF simulations indicate that the binding of GTP with the c.35G>A (p.G12D) mutant is less favorable compared with that of GTP with wild-type KRAS or the c.38G>A (p.G13D) mutant. Based on this observation, it is reasonable to hypothesize that c.38G>A (p.G13D) is similar to wild-type KRAS, and thereby the RAS-GTP hydrolysis reactions are preserved. By contrast, the *KRAS* mutation in codon 12 may impair the hydrolysis of GTP, leading the KRAS protein to take a permanent form. Our data make sense in light of the studies by Guerrero et al. [25], who demonstrated that *KRAS* codon 12 mutations confer a more aggressive tumor phenotype than codon 13 mutations by altering the threshold for the induction of apoptosis. Theoretically, codon 12-mutated KRAS remains in an active GTP-bound state longer than codon 13-mutated or WT KRAS. Herein, we have demonstrated that mutations in codon 13 can confer similar protein structure dynamics to WT KRAS. To gain better insight into why patients with metastatic colorectal cancer (mCRC) and the *KRAS* c.38G>A (p.G13D) mutation appear to benefit from anti-EGFR therapy, the role of the *KRAS* c.38G>A (p.G13D) mutation in mCRC needs to be further investigated.

### Conclusions

In this study, we have applied computational methods to understand the structural implications of c.35G>A (p.G12D) and c.38G>A (p.G13D) in the KRAS protein. Our findings underscore that the *KRAS* c.38G>A (p.G13D) mutation may exhibit similar behavior as *KRAS* WT. In summary, our data make sense in light of five recent studies [26]–[30], which demonstrated that *KRAS* codon 13 mutations, but not codon 12 mutations, conferred benefit from cetuximab therapy in advanced colorectal cancer.

## Supporting Information

Figure S1 **Protein dynamics simulation analysis.** RMSD plots of the WT (blue), G12D (red) and G13D (green) KRAS proteins with respect to the initial conformation during the course of MD simulations.
(TIF)

Figure S2 **The calculation of covariance matrices for WT, c.35G>A (p.G12D), and c.35G>A (p.G13D).** Covariance matrices calculated from MD trajectories for (A) WT, (B) G12D and (C) G13D.
(TIF)

Figure S3 **The second repeated molecular dynamics trajectories for:** (A) Comparison of the RMSD plots of the sensitive sites (P-loop, switch I and II regions) of WT, G12D and G13D structures with respect to the initial conformation during the course of the simulation; (B) the pocket distances between the mass center of residues 12–13 and the mass center of residues 32–34 for WT, G12D, and G13D, respectively.
(PDF)

Figure S4 **Analysis of atomic fluctuations in the second repeated MD simulations.** The structures of (A) WT, (B) G12D and, (C) G13D KRAS proteins are drawn in cartoon putty representations at the P-loop, switch I and II regions; blue represents the lowest and red the highest B-factor value. In addition, the size of the tube reflects the value of the B-factor, in that the larger the B-factor, the thicker the tube. The structures in the other regions are colored in white and displayed in cartoon tube representation, where the size of the tube is independent of the B-factors.
(PDF)

Figure S5 **The third repeated molecular dynamics trajectories for:** (A) Comparison of the RMSD plots of the sensitive sites (P-loop, switch I and II regions) of WT, G12D and G13D structures with respect to the initial conformation during the course of the simulation; (B) the pocket distances between the mass center of residues 12–13 and the mass center of residues 32–34 for WT, G12D, and G13D, respectively.
(PDF)

Figure S6 **Analysis of atomic fluctuations in the third repeated MD simulations.** The structures of (A) WT, (B) G12D and, (C) G13D KRAS proteins are drawn in cartoon putty representations at the P-loop, switch I and II regions; blue represents the lowest and red the highest B-factor value. In addition, the size of the tube reflects the value of the B-factor, in that the larger the B-factor, the thicker the tube. The structures in the other regions are colored in white and displayed in cartoon tube representation, where the size of the tube is independent of the B-factors.
(PDF)

Figure S7 **Prevalence of the KRAS gene mutation in CRC and distribution of KRAS mutational status in a Spanish population.** A total of 252 patients with mCRC confirmed at the Pathology Department of General Yagüe Hospital (Burgos, Spain) were included in the present study. Mutant KRAS in exon 2 was detected using a validated KRAS mutation kit (DxS Ltd, Manchester, United Kingdom) that identifies seven somatic mutations located in codons 12 and 13 using allele-specific real-time polymerase chain reaction. Central laboratory personnel validated the assays for their analytic and diagnostic performance, established acceptance criteria, included appropriate quality controls for each assay, and performed the KRAS analysis in a blinded fashion. The analysis was performed in an ABI Prism 7500 instrument (Applied Biosystems).
(TIF)
