# Supplementary figures and images for: Computational Analysis of KRAS Mutations: Implications for Different Effects on the KRAS p.G12D and p.G13D Mutations
Source: PLoS One. 2013 Feb 20;8(2):e55793. doi: 10.1371/journal.pone.0055793 (PMC3577811; doi:10.1371/journal.pone.0055793)

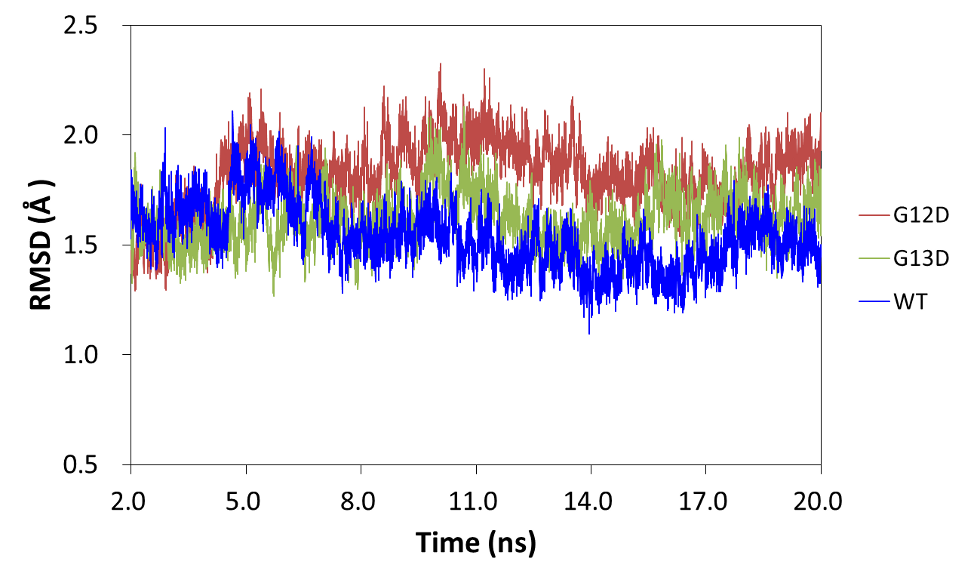

Supplement: Figure S1 — Protein dynamics simulation analysis. RMSD plots of the WT (blue), G12D (red) and G13D (green) KRAS proteins with respect to the initial conformation during the course of MD simulations. (TIF) [file pone.0055793.s001.tif]

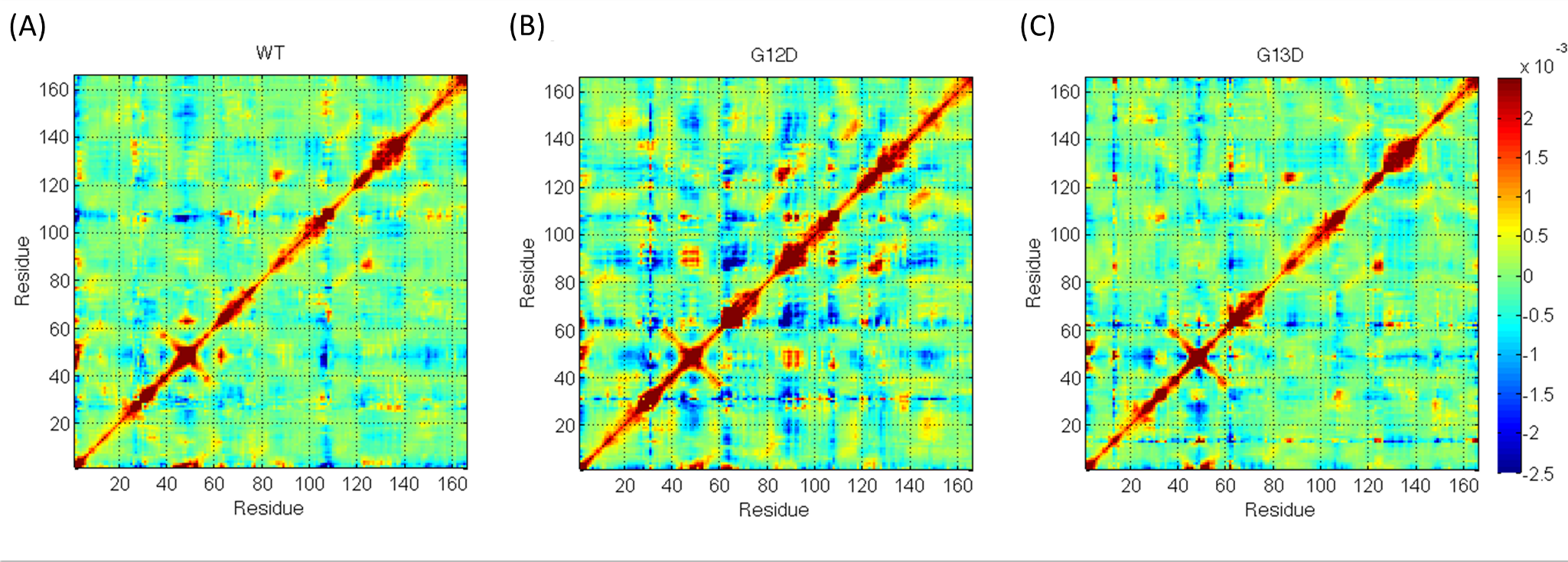

Supplement: Figure S2 — The calculation of covariance matrices for WT, c.35G>A (p.G12D), and c.35G>A (p.G13D). Covariance matrices calculated from MD trajectories for (A) WT, (B) G12D and (C) G13D. (TIF) [file pone.0055793.s002.tif]

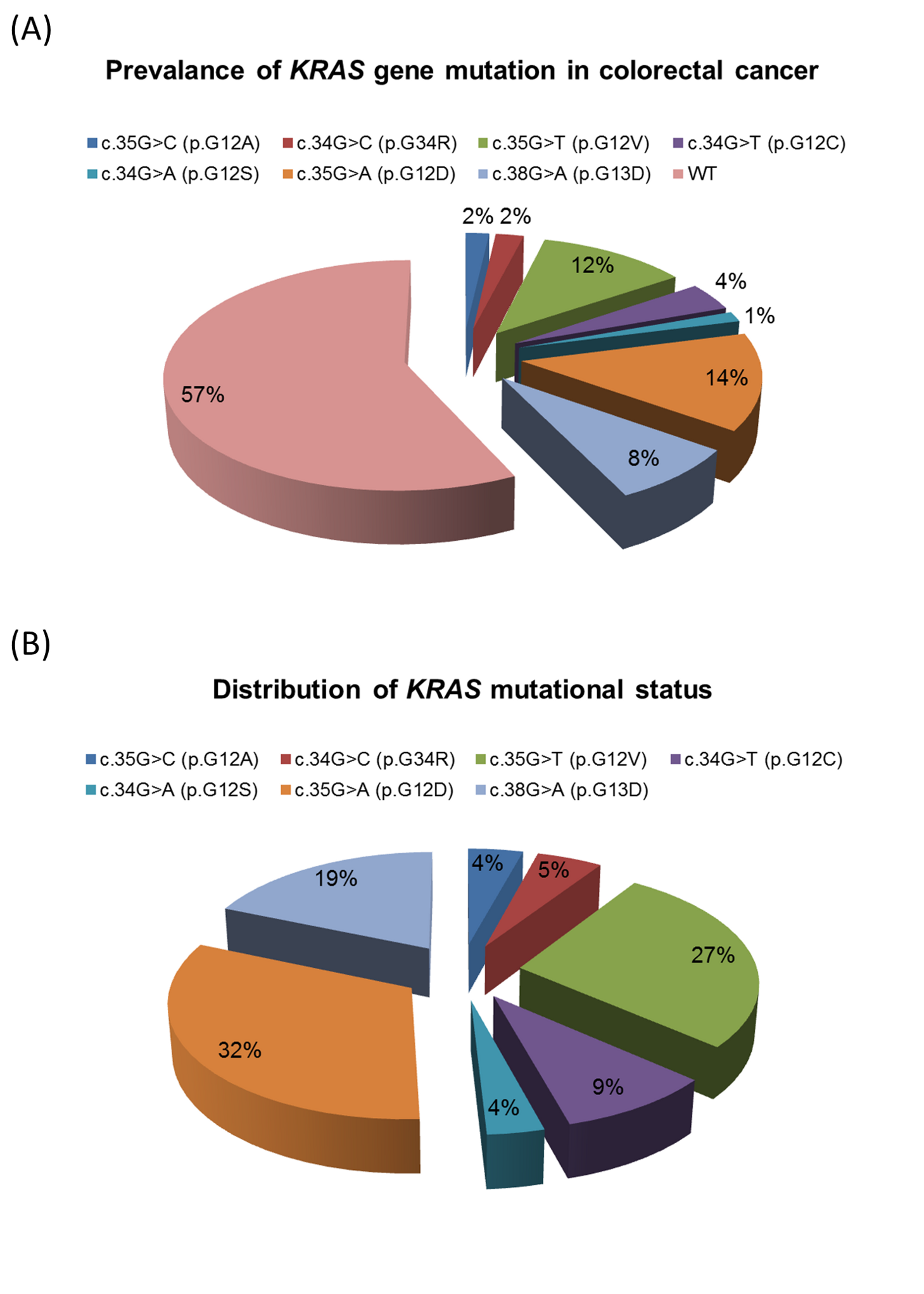

Supplement: Figure S7 — Prevalence of the KRAS gene mutation in CRC and distribution of KRAS mutational status in a Spanish population. A total of 252 patients with mCRC confirmed at the Pathology Department of General Yagüe Hospital (Burgos, Spain) were included in the present study. Mutant KRAS in exon 2 was detected using a validated KRAS mutation kit (DxS Ltd, Manchester, United Kingdom) that identifies seven somatic mutations located in codons 12 and 13 using allele-specific real-time polymerase chain reaction. Central laboratory personnel validated the assays for their analytic and diagnostic performance, established acceptance criteria, included appropriate quality controls for each assay, and performed the KRAS analysis in a blinded fashion. The analysis was performed in an ABI Prism 7500 instrument (Applied Biosystems). (TIF) [file pone.0055793.s007.tif]
