# Supplementary material for: Computational Analysis of KRAS Mutations: Implications for Different Effects on the KRAS p.G12D and p.G13D Mutations
Source: PLoS One. 2013 Feb 20;8(2):e55793. doi: 10.1371/journal.pone.0055793 (PMC3577811; doi:10.1371/journal.pone.0055793)
Supplement: Figure S5 — The third repeated molecular dynamics trajectories for: (A) Comparison of the RMSD plots of the sensitive sites (P-loop, switch I and II regions) of WT, G12D and G13D structures with respect to the initial conformation during the course of the simulation; (B) the pocket distances between the mass center of residues 12–13 and the mass center of residues 32–34 for WT, G12D, and G13D, respectively. (PDF) [file pone.0055793.s005.pdf]

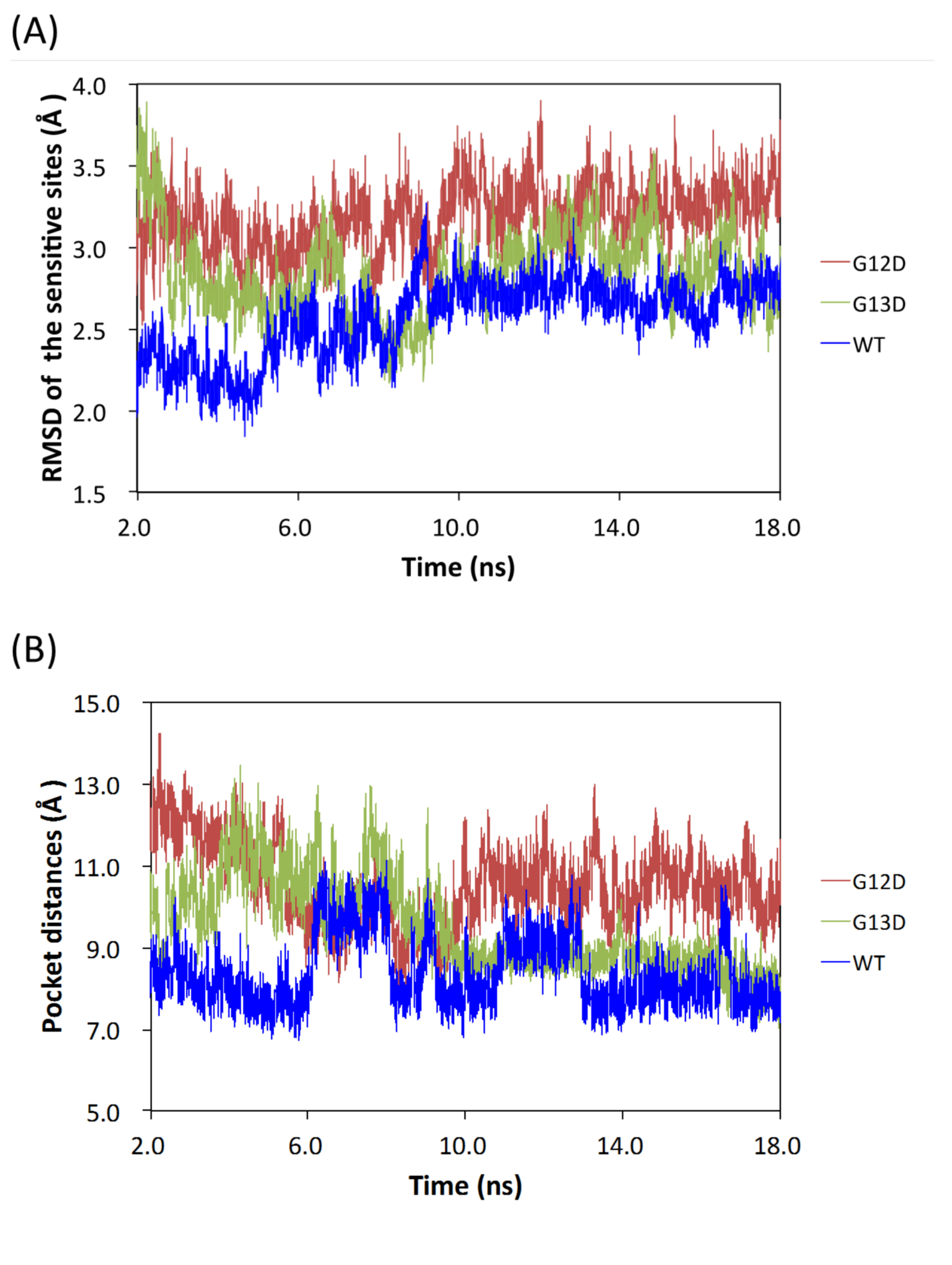

Figure S5 – The third repeated molecular dynamics trajectories for:

(A) Comparison of the RMSD plots of the sensitive sites (P-loop, switch I and II regions) of WT, G12D and G13D structures with respect to the initial conformation during the course of the simulation; (B) the pocket distances between the mass center of residues 12-13 and the mass center of residues 32-34 for WT, G12D, and G13D, respectively.
