# Supplementary material for: Computational Analysis of KRAS Mutations: Implications for Different Effects on the KRAS p.G12D and p.G13D Mutations
Source: PLoS One. 2013 Feb 20;8(2):e55793. doi: 10.1371/journal.pone.0055793 (PMC3577811; doi:10.1371/journal.pone.0055793)
Supplement: Figure S6 — Analysis of atomic fluctuations in the third repeated MD simulations. The structures of (A) WT, (B) G12D and, (C) G13D KRAS proteins are drawn in cartoon putty representations at the P-loop, switch I and II regions; blue represents the lowest and red the highest B-factor value. In addition, the size of the tube reflects the value of the B-factor, in that the larger the B-factor, the thicker the tube. The structures in the other regions are colored in white and displayed in cartoon tube representation, where the size of the tube is independent of the B-factors. (PDF) [file pone.0055793.s006.pdf]

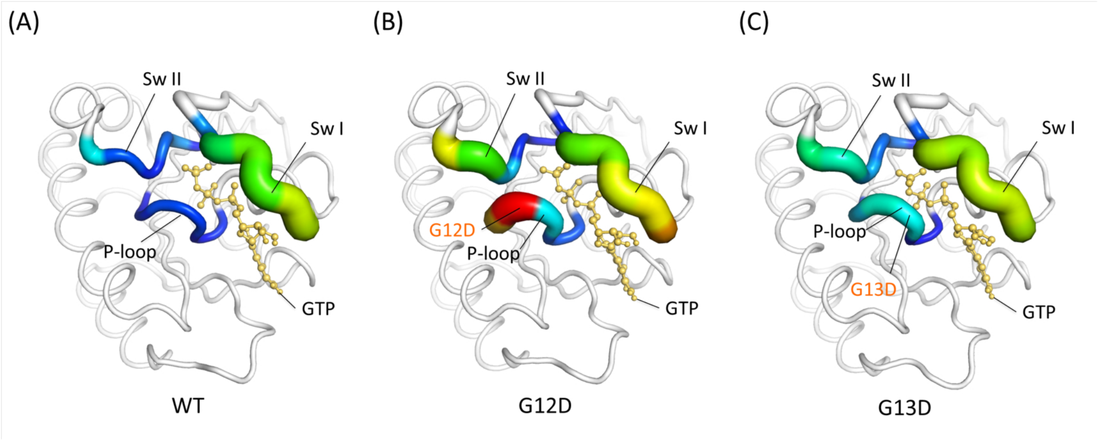

Figure S6 - Analysis of atomic fluctuations in the third repeated MD simulations.

The structures of (A) WT, (B) G12D and, (C) G13D KRAS proteins are drawn in cartoon putty representations at the P-loop, switch I and II regions; blue represents the lowest and red the highest B-factor value. In addition, the size of the tube reflects the value of the B-factor, in that the larger the B-factor, the thicker the tube. The structures in the other regions are colored in white and displayed in cartoon tube representation, where the size of the tube is independent of the B-factors.
